# Removal of Antibiotics and Nutrients by Vetiver Grass (*Chrysopogon zizanioides*) from a Plug Flow Reactor Based Constructed Wetland Model

**DOI:** 10.3390/toxics9040084

**Published:** 2021-04-15

**Authors:** Saumik Panja, Dibyendu Sarkar, Zhiming Zhang, Rupali Datta

**Affiliations:** 1 Department of Civil, Environmental and Ocean Engineering, Stevens Institute of Technology, Hoboken, NJ 07030, USA; saumikpanja@gmail.com (S.P.); zzhan100@stevens.edu (Z.Z.); 2 Department of Biological Sciences, Michigan Technological University, Houghton, MI 49931, USA; rupdatta@mtu.edu

**Keywords:** vetiver grass, phytoremediation, constructed wetland, antibiotics, nutrients

## Abstract

Overuse of antibiotics has resulted in widespread contamination of the environment and triggered antibiotic resistance in pathogenic bacteria. Conventional wastewater treatment plants (WWTPs) are not equipped to remove antibiotics. Effluents from WWTPs are usually the primary source of antibiotics in aquatic environments. There is an urgent need for cost-effective, environment-friendly technologies to address this issue. Along with antibiotics, nutrients (nitrogen and phosphorus) are also present in conventional WWTP effluents at high concentrations, causing environmental problems like eutrophication. In this study, we tested vetiver grass in a plug flow reactor-based constructed wetland model in a greenhouse setup for removing antibiotics ciprofloxacin (CIP) and tetracycline (TTC), and nutrients, N and P, from secondary wastewater effluent. The constructed wetland was designed based on a previous batch reaction kinetics study and reached a steady-state in 7 days. The measured concentrations of antibiotics were generally consistent with the modeling predictions using first-order reaction kinetics. Vetiver grass significantly (*p* < 0.05) removed 93% and 97% of CIP and TTC (initial concentrations of 10 mg/L), simultaneously with 93% and 84% nitrogen and phosphorus, respectively. Results show that using vetiver grass in constructed wetlands could be a viable green technology for the removal of antibiotics and nutrients from wastewater.

## 1. Introduction

Pharmaceutical compounds are major emerging contaminants that persist in soil and aquatic environments due to their prolonged bioactivity [1]. Antibiotics in the environment are considered a major concern since they trigger antimicrobial resistance in bacteria and impact the ecosystem in various ways. Recently, the European Union and the United States conducted a nationwide surface water sampling study which revealed the existence of several antibiotics, such as macrolides and quinolones, in aquatic ecosystems [2]. Most of the antibiotics are highly polar and have low volatility which is optimal for activity in the human physiological system. A few lipophilic varieties of antibiotics also exist which facilitates their trophic transfer in aquatic ecosystems [3,4]. Among all the synthetic antibiotics, fluoroquinolones have a significant market share as they are widely prescribed by physicians. Ciprofloxacin (CIP) is one of the major fluoroquinolone antibiotics that is used in human health care and veterinary practices. Tetracycline (TTC) is a naturally sourced antibiotic that is obtained from *Streptomyces sp.* and used to treat diseases such as malaria, rosacea, chlamydia, etc. [5,6,7]. TTC is also administered to animals as a growth promoter in concentrated animal feeding operations. Both CIP and TTC are non-volatile solids and contain acidic and basic functional groups. Depending on the pH, they can exist as anions, cations, or zwitterions in the aqueous phase. Their solubility and octanol-water distribution ratio also depend on the solution pH [8,9,10].

Due to the alarming increase of pharmaceutical compounds, personal care products, and endocrine disruptors in the environment, many nations are considering different countermeasures and implementing regulatory actions to control their negative environmental impact. Wastewater treatment plants (WWTPs) that receive effluents from pharmaceutical industries and hospitals serve as major contributors to these emerging contaminants in the aquatic environment. Conventional WWTPs not only release antibiotics through their effluents but also serve as point sources for nutrient (e.g., nitrogen and phosphorus) discharge, causing environmental problems like eutrophication. Constant nutrient loadings into surface water pose an increasing threat to the aquatic ecosystem [11,12]. Increasing urbanization has also resulted in increased nitrate leaching into groundwater, which constitutes a major ecological and human health risk [13]. Other drawbacks of conventional WWTPs include a significant consumption of energy (e.g., activated sludge processes for the removal of organics) and the disposal of the residual sludge, which impose a financial burden on the operation and management (O&M) cost [14,15,16]. Some physicochemical processes, including advanced oxidation via ozonation [17] or ferrous-activated persulfate [18], photodegradation [19], and adsorption [20,21], have been tested for antibiotics removal, but these treatments are cost-intensive and may generate toxic derivatives from the parent antibiotic compounds.

In contrast, the application of phytoremediation for contaminant removal has become popular due to its ability to remove a diverse range of organic and inorganic contaminants from soil and water with a variety of advantages, such as low O&M cost, simple design, and low or no environmental impacts [4,22,23,24,25,26,27,28]. Phytoremediation comprises a series of unique processes such as absorption, adsorption, degradation, and biotransformation of contaminants [29,30,31,32]. Not only do the plants decontaminate pollutants but also the exudates released from plant root systems catalyze microbial growth and facilitate exocellular biotransformation of organic contaminants.

The ideal candidates for phytoremediation are macrophytes that can tolerate, and accumulate/degrade, toxic pollutants in their tissues. Vetiver grass (*Chrysopogon zizanioides*) is one of the best-suited species for nutrient removal [33,34]. Being a member of the grass family, vetiver is a fast-growing, high biomass tropical perennial grass that has a massive root system. Vetiver grass was also reported to withstand extreme conditions like acidic environment (pH = 0.6), concentrated nutrients (nitrate concentrations up to 350,000 mg/L), freezing temperature (5 °F or −15 °C), and drought (up to 15 months) [33,34]. The multipurpose applications of vetiver grass include prevention of soil erosion [35,36], protection of river banks [37,38,39], removal of lead from soil [40,41,42,43], management of acid mine drainage impacted soil and water [44,45,46], removal of explosives from soil and water [44,47,48,49,50], decontamination of heavy metals from landfill leachate [51,52], removal of antibiotics from soil and water [53,54,55], and removal of nutrients (nitrogen and phosphorus) [56], etc. Our previous batch studies have shown that vetiver grass is not only capable of removing antibiotics (TTC and CIP) from wastewater but also degrading and metabolizing antibiotic molecules [54,57,58,59].

Plug flow treatment is an efficient technology used where space is a prime concern. The residence time can be efficiently manipulated in a plug flow reactor (PFR) to achieve contaminant removal. Constructed wetlands, as a sustainable and cost-effective technology, exhibit great potential to treat domestic, agricultural, and industrial wastewater. However, phytoremediation treatment processes for emerging contaminants (e.g., antibiotics) via vegetated constructed wetland are often considered as a ‘black box’ due to the lack of proper scientific investigation [60]. Thus, the major objective of this study was to investigate the efficiency of a vegetated (using vetiver grass) constructed wetland in the form of a PFR to remove CIP and TTC from secondary wastewater effluent. In addition to antibiotics, our objectives were to characterize the removal of nutrients (i.e., nitrogen and phosphorous) and carbon using the PFR.

## 2. Materials and Methods

### 2.1. Reagents

Reagent grade (≥98%) CIP and TTC were procured from Sigma Aldrich (Millipore-Sigma, St. Louis, MO, USA). Trace metal grade ortho-phosphoric acid (H_3_PO_4_, CAS 7664-38-2), HPLC grade methanol (CAS 67-56-1), and acetonitrile (CAS 75-05-8) were purchased from Fisher Scientific (Fairlawn, NJ, USA). Anhydrous oxalic acid (C_2_H_2_O_4_, CAS 144-62-7) was purchased from Acros Organics (Fairlawn, NJ, USA).

### 2.2. Wastewater Collection

The secondary treatment run-off effluent was collected from the secondary clarifier at Joint Meeting Wastewater Treatment Plant (Capacity: 85 million gals or 322 million L per day) located at Elizabeth, NJ, USA. The plant serves the urban areas of two majorly populated counties (Essex and Union) in NJ, USA. Approximately 2300 L of wastewater were collected in 32-gallon polyethylene containers. The wastewater effluent was transported immediately to the laboratory and stored in 4 °C. Primary characterization for wastewater was performed on the same day of collection.

### 2.3. Experimental Design

Vetiver slips (8–10 inches long), supplied by Agriflora Tropicals in Puerto Rico, were initially potted in a greenhouse environment (25 °C and 14 h photoperiod) using a commercially available potting mixture for two months. Then, the plants were taken out of the pots and the roots were thoroughly washed to remove soil particles. Roots and shoots were trimmed using a sterilized pruning tool, and the roots were submerged into 0.5× Hoagland’s solution for acclimatization (for 20 days) in a Thermo Scientific Precision plant growth chamber.

### 2.4. Constructed Wetland Setup

**Figure 1:**
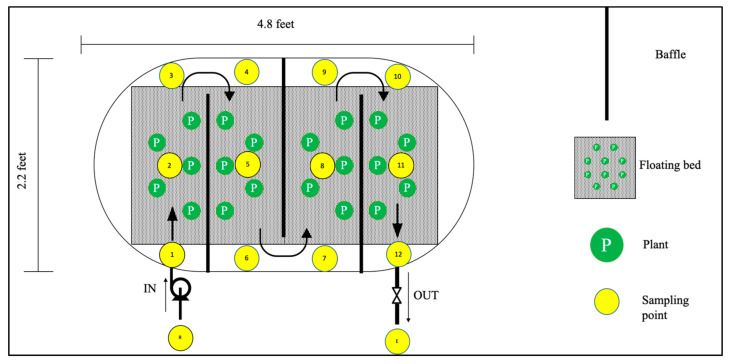
Schematic diagram of the constructed wetland.

**Figure 2:**
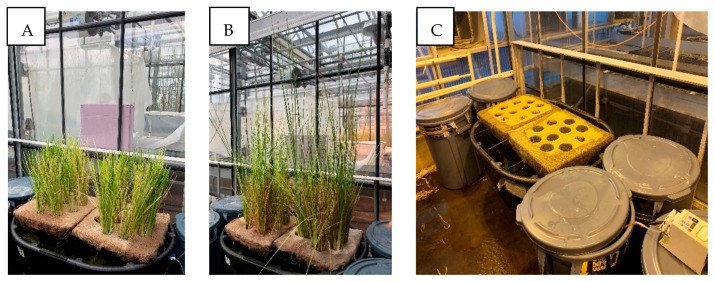
The vegetated (**A**,**B**) and plant-free control (**C**) plug flow reactor (PFR)-based constructed wetland system. Vetiver grass before the start of the experiment (**A**) and the growth of grass after 15 days (**B**).

Two 150-gallon (0.57 m^3^) tanks were used for setting up a mesocosm scale constructed wetland. Acrylic sheet baffles (1/8 inch or 0.32 cm in width) were used to make partitions in the tank (Figure 1). The inner dimension of the tank was measured to design 3 baffles that can fit edge to edge inside the tank. Then, the acrylic sheets were cut accordingly in a machine shop. Some extra portions were also cut to maintain the cross-sectional area of the PFR (Figure 1). The baffles were inserted and sealed with non-toxic inert silicone-based aquarium glue so that water can only pass through the cross-sectional area. Water inlet and outlet (Figure 1) were installed in each tank using lead-free quick-connect straight valves (1/4-inch or 0.64 cm OD). PVC clear vinyl tubing (1/4-inch or 0.64 cm OD) was used for supplying water from the storage tank to the wetland. A two-channel Ismatec peristaltic pump (ISM 832, Cole-Parmer, IL, USA) was used to pump the wastewater from the storage to the wetland tank. Customized floating beds manufactured from inert materials were purchased from Floating Islands West LLC (Mokelumne Hill, CA, USA) and used to setup the vegetated macrophyte bed in the wetland tank. The beds were modified by cutting holes small enough to insert the plant roots through them. Plants (Figure 2A) were weighed (4% of the volume) and equally distributed in the floating bed panels. The control tank contained only the baffles and floating beds (Figure 2C). The overall reaction was assumed in PFR mode owing to the evenly distributed vetiver plants in the system that are expected to have similar reactions with chemicals along the water path. The feasibility of this PFR model was assessed by comparing it with experimental data for CIP and TTC removal. The hydraulic retention time (HRT) was determined (θ = 7 days) using the first-order reaction kinetics from previous batch studies [57]. The wastewater flow rate was set to 50 mL/min and the hydraulic loading rate (HLR) was maintained at 34 mL/m^2^-min. The elevation of the tank was adjusted in a way to approximately match the flow rate of inflow and outflow of the water. Several dry runs were performed using tap water to optimize the physical parameters before starting the actual experiment. Concentrated stock solutions of CIP and TTC were added (10 mg/L) in the feed tank and stirred overnight to dissolve. The initial concentrations of both antibiotics were checked using HPLC.

### 2.5. Analytical Methods

#### 2.5.1. Physico-Chemical Parameters

Physico-chemical parameters like temperature, pH, and electrical conductivity were measured using a multi-parameter water quality sonde (YSI-6820, YSI, OH, USA). The pH meter was calibrated each time using standard solutions before sample measurement.

#### 2.5.2. Chlorophyll Content

Chlorophyll (Chl) content of the plant leaves was determined according to Liu et al. (2013) [61]. In brief, fresh plant leaves were homogenized in 5 mL of 80% (*v*/*v*) aqueous acetone followed by filtration. Then, the absorbance (663 and 645 nm) of the filtrate was measured using Citation 3, Biotek^®^ microplate reader. The following equations were used to determine Chl A and Chl B content, respectively [62].

(1) $$Chl\;A=0.00127Abs_{663}-0.00269Abs_{645}\;$$

(2) $$Chl\;B=0.0229Abs_{645}-0.00468Abs_{663}$$

#### 2.5.3. Antibiotics

Antibiotic content in the wastewater were analyzed according to Panja et al. (2020) [57]. Briefly, CIP and TTC content in samples were analyzed using Agilent 1260 HPLC system (Agilent, CA, USA) equipped with a photodiode array (PDA) detector and a 1260 series autosampler. For both antibiotics, a Hypersil gold C18 column (150 × 4.6 mm, 5 μm) (Thermo Scientific, MA, USA) with a corresponding Hypersil gold guard column (10 × 4 mm, 5 μm) were used. The acidic (pH = 3) mobile phase used for CIP detection contained orthophosphoric acid and acetonitrile (80:20 *v*/*v*). The wavelength of the UV detector was set at 360 nm. The mobile phase for TTC samples elution contained 0.01 M oxalic acid: acetonitrile: methanol (150:20:20 by volume). The flowrate for both TTC and CIP analysis was maintained at 1.5 mL/min and the sample injection volume was set at 25 μL. The HPLC was calibrated using standard solutions of CIP and TTC. Method detection limits for CIP and TTC were 10 ug/L and 0.1 mg/L, respectively.

#### 2.5.4. Total Nitrogen, Total Phosphorus, and Chemical Oxygen Demand

Total nitrogen (TN), total phosphorus (TP), and chemical oxygen demand (COD) of all samples were measured using Hach test kits (Hach, CO, USA). A Hach 36 chamber digestion block was used to digest water samples for the determination of COD. A DR 6000 UV Vis spectrophotometer (Hach, CO, USA) was used for all the analyses. Detection limits for TN and TP were 0.1 mg/L and 0.06 mg/L, respectively.

#### 2.5.5. Statistical Analysis

JMP Pro 11 (SAS Inc., Cary, NC, USA) was used for all statistical analyses. Q-tests were performed on all data to eliminate possible outliers at the 95% confidence interval. All samples from each treatment were taken in triplicates. Tukey Kramer HSD test was done to determine significant differences among treatment means.

## 3. Results

### 3.1. Wastewater Characterization

The physical and chemical analysis of the wastewater revealed that both nitrogen and phosphorous were present as primary pollutants at concentrations of 14.4 and 12.3 mg/L, respectively (Table 1). The water was mildly turbid upon collection 13.4 NTU (Nephelometric Turbidity Units). The activated sludge treatment process removes most of the organic load from wastewater. The secondary wastewater effluent contained 62 mg/L chemical oxygen demand (COD) and 21 mg/L total organic carbon (TOC). The pH of the collected wastewater was neutral at 6.8 and the electrical conductivity was 801 μS/cm.

### 3.2. Constructed Wetland Experiment

**Figure 3:**
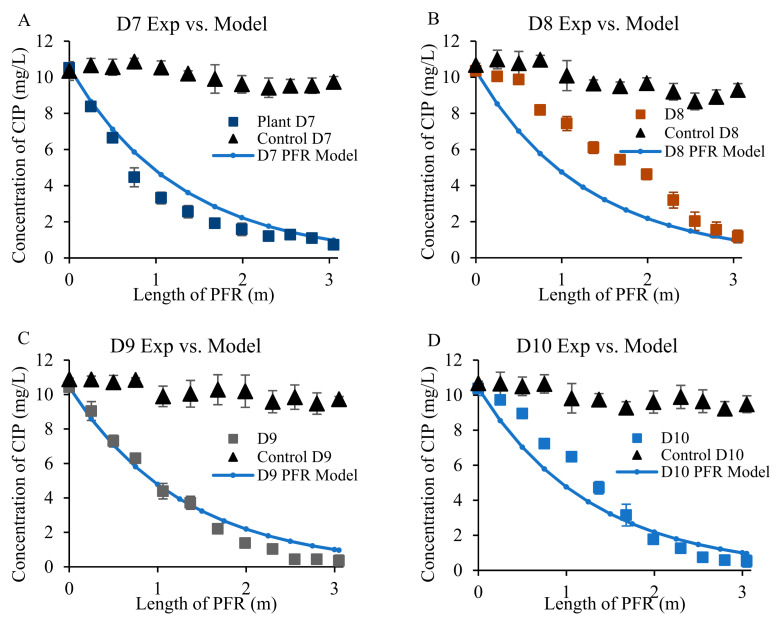
Removal of ciprofloxacin (CIP) according to predicted and experimental data for day 7 (D7, **A**), Day 8 (D8, **B**), Day 9 (D9, **C**), and Day 10 (D10, **D**).

**Figure 4:**
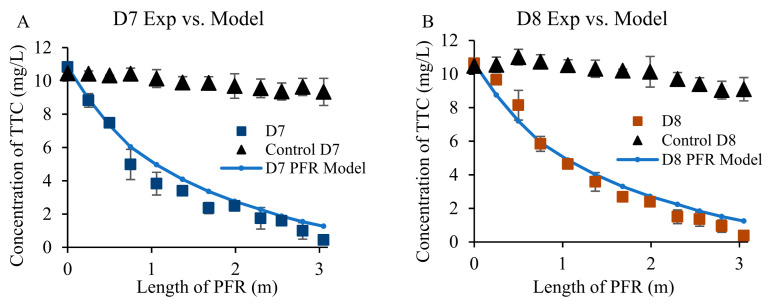

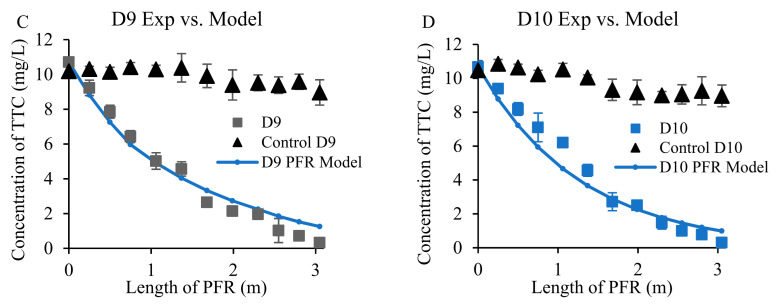
Removal of tetracycline (TTC) according to predicted and experimental data for day 7 (D7, **A**), Day 8 (D8, **B**), Day 9 (D9, **C**), and Day 10 (D10, **D**).

**Figure 5:**
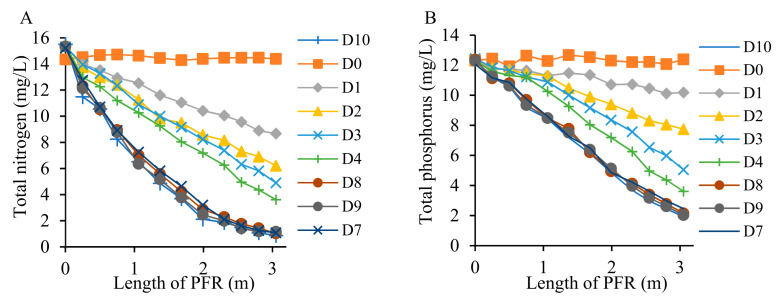
Removal of total nitrogen (TN) (**A**) and total phosphorus (TP) (**B**) in pre- and post- steady-state conditions from vegetated constructed wetland (D0-D10).

**Figure 6:**
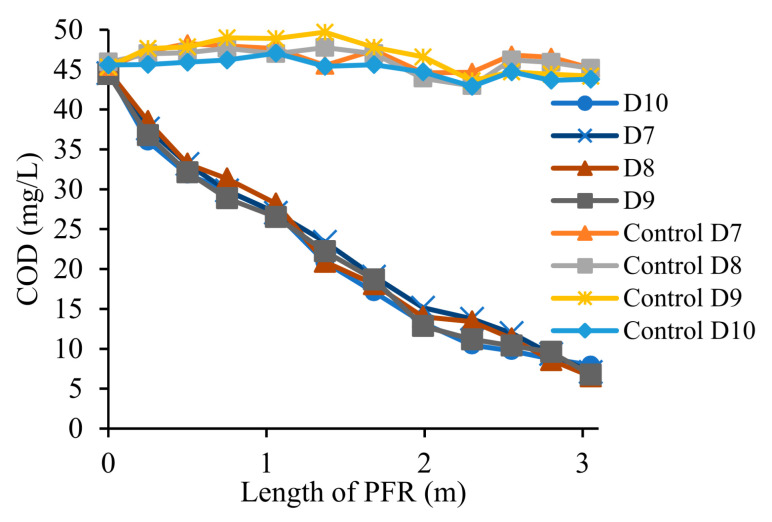
Removal of chemical oxygen demand (COD) in steady-state condition from both vegetated and plant-free constructed wetland (D7-D10).

**Figure 7:**
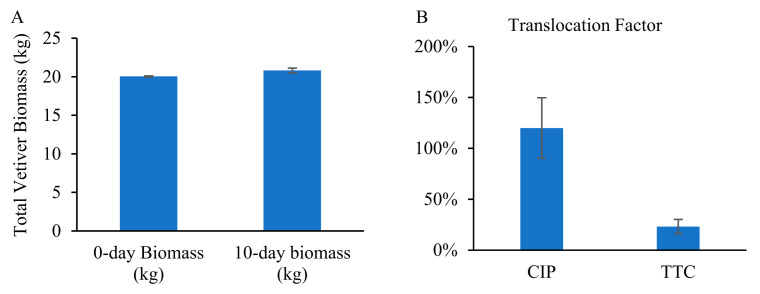
Comparison of initial and final total biomass of vetiver grass during the experimental period (**A**) and root to shoot translocation factor of CIP and TTC (**B**).

Our previous studies have demonstrated rapid removal of antibiotics CIP and TTC during the first 5–7 days of the treatment [57]. We determined that the initial drop in the antibiotic concentration followed first-order reaction kinetics (reaction kinetic constant k = 0.16 day^−1^) [58]. Depending on this kinetic constant, we modulated the flow of influent so that the HRT of the reactor remained at 7 days. As per our model prediction, the reactor was estimated to enter a steady-state condition by day 7, which was demonstrated by a preliminary simulation experiment and the relatively steady concentrations of antibiotics, nutrients, and COD in the effluent on and after day 7 in this study (Figure 3, Figure 4, Figure 5 and Figure 6). By installing the baffles, we elongated the influent path length to 3.05 m (Figure 1). Vetiver grass showed extensive growth (both root and shoot) in the wastewater-fed hydroponic setup (Figure 2B and Figure 7).

### 3.3. Removal of Antibiotics from Wastewater

The constructed wetland reached a steady-state after 7 days, confirmed by the relatively stable concentrations of antibiotics, nutrients, and COD in the effluent. During the steady-state condition, the difference between influent and effluent in terms of antibiotic (CIP and TTC) concentrations was statistically significant (*p* < 0.05). On average, vetiver grass removed 93% and 97% of the initial CIP and TTC content (10 mg/L each), respectively, across the length of the PFR (Figure 3 and Figure 4).

A simulation of CIP and TTC distributions using the ideal PFR model was compared with experimental measurements to illustrate the reaction status in the constructed wetland. The simulated concentration at each sampling port is calculated by Equation (3):

(3) $$C_{model}=C_{0}\times e^{-kt}$$

where *C_model_* (mg/L) is the simulated concentration for CIP or TTC, *C*_0_ (mg/L) is the concentration of CIP or TTC in the influent (i.e., 10 mg/L for both), *k* (day^−1^) is the reaction kinetic constant 0.16 day^−1^, and *t* (day) is the absolute residence time for each sampling port.

Results showed that there was a general match between the experimental and predictive model data points (Figure 3 and Figure 4), except for only one CIP data set obtained on day 8 (Figure 3B) which showed an average of 20% deviation between simulated and experimental values. The overall PFR simulation for TTC removal was better than that of CIP. This may be caused by the difference in the vetiver’s tolerance to the two antibiotics. The deviation of experimental data from the predicted value on day 8 could be related to plant stress to CIP. Ideally, the plant-free control reactor was optimized to reach a steady-state within a week, which was similar to the vegetated constructed wetland. The equality of variance was tested between control and treatment. For each day in a steady-state condition (Day 7 to Day 10, indicated as D7–D10), the variance between control and treatment was not equal for all comparisons at a 99% confidence level. Welch’s two-sample t-test, which was performed to test the difference in mean between control and treatment, revealed that the two populations (unplanted control and vetiver treatment) means were also unequal at a 1% level of significance. In the plant-free control setup, the CIP and TTC concentrations dropped by 11% and 15%, respectively, during steady-state.

### 3.4. Removal of Nutrients from Wastewater

Figure 5 shows that the concentrations of both TN and TP in the effluent dropped gradually from day 0 to day 7. The reactor reached a steady-state condition on day 7, as those concentrations in each sampling port as well as in the effluent remained stable after that time. Similar to antibiotics removal, vetiver significantly (*p* < 0.05) removed TN and TP from the PFR-based constructed wetland. In steady-state conditions, 93% of TN and 84% of TP were depleted from secondary wastewater effluent (Figure 5). Figures S1 and S2 show the removal of TN and TP in comparison to our model prediction. As seen in the case of antibiotics, there was a good match between the experimental and predictive model data points.

### 3.5. Removal of COD from Wastewater

As the antibiotic content contributes toward organic carbon, the COD of the antibiotic spiked wastewater was approximately 10% greater than the actual raw secondary wastewater effluent. Vetiver grass successfully consumed 84% of total COD from the wastewater during steady-state, along with the removal of antibiotics and nutrients. During steady-state (D7–D10), there were no significant (*p* < 0.05) differences in the data points obtained from both the vegetated constructed wetland and the plant-free control (Figure 6). The negligible COD decline (up to 4%) in the plant-free control PFR may be attributed to the growth of antibiotic-resistant organisms.

### 3.6. Chlorophyll Content and Biomass Development

**Figure 8:**
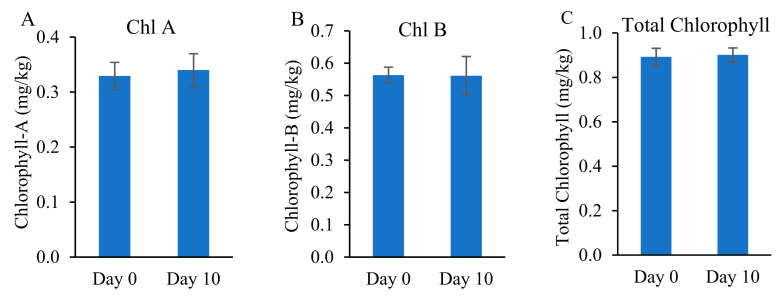
Comparison of the initial and final content of chlorophyll-A (**A**), chlorophyll-B (**B**), and total chlorophyll (**C**) in vetiver grass.

Figure 8 shows the contents of Chl A, Chl B, and total Chl in vetiver grass. Chl A content increased by 3% by the end of the 10-day experimental period and Chl B content remained the same. We did not observe any significant (*p* < 0.05) difference in the chlorophyll content before and after the experimental period.

## 4. Discussion

The results obtained in this study show that vetiver grass in a constructed wetland setup was able to remove antibiotics and nutrients with high efficiency. The removal kinetics of these contaminants followed the PFR model predictions and reached a steady-state after 7 days. This result is similar to our previous studies, where we observed biphasic antibiotics (CIP and TTC) removal by vetiver grass [57]. The rapid uptake of antibiotics up to 5–7 days was followed by a slower removal subsequently [57]. We determined that the initial drop in the antibiotic concentration followed first-order reaction kinetics (reaction kinetic constant k = 0.16 day^−1^) [58].

Previous literature reported that the removal of CIP and TTC was mainly through adsorption by plant roots, and translocation from roots to shoots may be limited [63,64]. The massive root system of vetiver grass contributed to the high removal efficiencies in both CIP and TTC. The removal trend for CIP and TTC is consistent with our previous studies that vetiver grass showed more affinity towards the removal of TTC compared to the CIP [57,58]. This difference could be due to the difference in how CIP and TTC impact the vetiver, causing variation in uptake and accumulation of these antibiotics [54,59,63]. The overall performance of vetiver grass in a PFR-based constructed wetland system was better than in our previous batch study. Specifically, our previous hydroponic batch experiments showed vetiver grass removed only 71% of CIP and 90% of TTC after 7 days [56]. The flow condition in a PFR system promotes better contact of the antibiotics with vetiver roots, thus facilitating improved removal compared to batch reactions. Another reason for improved antibiotic removal could be the cumulative/bulk effect.

The vetiver plants showed good growth and survived well until the end of the experiment, although a few common stress symptoms (e.g., mild chlorosis and drying leaf tips) were observed at initial exposure to the CIP and TTC-spiked wastewater. The growth of plants could be explained by the presence of nutrients (NO_3_^−^-N at 29 mg/L and PO_4_^3−^-P at 10.5 mg/L). In our previous study, vetiver showed visible physical stress symptoms at a CIP concentration of 10 mg/L [59], while no significant stress symptoms were seen when vetiver was exposed to an even higher concentration of TTC (75 mg/L) [54]. Higher stress on vetiver was also induced by CIP than TTC in the current study, which may be caused by higher translocation factor for CIP, which induced more CIP accumulation in vetiver shoots. Thus, the experimental removal of TTC was more consistent with the predicted removal. However, vetiver was more sensitive to CIP concentrations and other minor variations in experimental conditions (e.g., temperature and availability of light, [65,66]), which induced deviations between experimental and predicted outcomes.

Since the uptake of contaminants can affect the overall health and growth of the plant, which could be indicated by Chl content in plant shoot tissue [61,67], it is implied that the overall health of vetiver grass was not significantly influenced in this study. In comparison, our previous studies have shown that CIP negatively affects the chlorophyll content of vetiver grass [59]. This may be a cumulative effect of CIP-induced stress as it also affected the total protein content of vetiver root and shoot and triggered activation of stress enzymes [59]. Rydzyński et al. (2017) also reported a significant decrease of Chl in the leaves of Yellow Lupine, especially in new leaves, when exposed to CIP and TTC [68]. CIP and TTC induce chl decay and a decrease in chl concentration. However, in this study, the chl content remained relatively steady, and the biomass of the plants increased by 4% within 10 days without significant physical stress symptoms. These observations indicate that vetiver grass may tolerate antibiotic stress better when plant density is high when compared to individual plants that were used in our previous report in Panja et al. [59], and Rydzyński et al. [58,68].

The decline in CIP and TTC concentrations in the plant-free control setup may have been caused by adsorption, and bacterial degradation. Golet et al. (2003) reported the removal of antibiotics (e.g., CIP) associated with suspended solids in wastewater [69]. Lou et al. (2018) demonstrated the adsorption of TTC on suspended organic matters in swine wastewater [70]. Thus, with turbidity at 13.4 NTU and electrical conductivity of 801 μS/cm in the wastewater effluent, the loss of CIP and TTC in the plant-free control setup may be induced by adsorption onto solids and then precipitation, which would have been removed by filtration before the antibiotics were quantified. Another possibility is bacterial degradation. Antibiotic-resistant bacteria may exist in the wastewater effluent used for this research [71]. Bacteria in wastewater treatment facilities may also become antibiotic-resistant through horizontal gene transfer [72]. Therefore, the decrease in the antibiotic concentration in the control setup could be attributed to microbial degradation. Since TTC is more widely applied and has been in use much longer than CIP, TTC-resistant bacteria may be more abundant than CIP-resistant bacteria [58,72], leading to more TTC degradation than CIP.

In our study, vetiver was able to remove significant levels of TN and TP. Nitrogen and phosphorous removal from wastewater by phytoremediation have been widely reported. The removal efficiencies by plants are highly dependent on the plant species and the co-existing microorganisms in wastewater. Ojoawo et al. (2015) tested *Canna generalis* reeds for the removal of nitrate and phosphate in domestic wastewater and found only 52% and 9% of these two contaminants were removed, respectively [73]. Recently, Nizam et al. (2020) compared five plants (*Centella asiatica*, *Ipomoea aquatica*, *Salvinia molesta*, *Eichhornia crassipes*, and *Pistia stratiotes*) for their capacity to remove nitrogen and phosphorous [74]. Although up to 98% of nitrogen was removed by *Centella asiatica* and up to 98% of phosphorous was removed by *Eichhornia crassipes*, there were significant differences in performance among the five species. In some cases, other contributors (e.g., microorganism degradation and solid adsorption/precipitation) may work more efficiently than plants. As described by Zhang et al. (2007), plants contributed to about 22% and 33% for TN and TP removal, respectively, while unplanted control experiments reached over 50% for both TN and TP [75]. Song et al. (2011) also reported the important role of microorganisms, along with macrophytes, in removing nutrients from water matrices [76]. In this research, high concentrations (10 mg/L) of two broad-spectrum antibiotics were spiked in the reactor influent, so the growth of most microorganisms was possibly inhibited in the reactor and vetiver grass contributed significantly to the TN and TP consumption, as only 7% of TN and 6% of TP were consumed in the plant free control reactor. The small decline in the control reactor could be due to the adsorption to solids and a small amount of degradation by antibiotic-resistant bacteria. Vetiver grass has been reported to remove nitrogen and phosphorous with high efficiency [49,57,77]. It can tolerate high concentrations of nutrients before significant stress symptoms are visible in the plant [49].

The high removal efficiencies for TN and TP observed in this study are consistent with previous studies on nutrient removal from wastewater by vetiver grass [49,57]. Upon reaching the steady-state condition, there were no significant differences in the removal kinetics of TN and TP. Wang and Sample (2014) reported a similar study where pickerelweed (*Pontederia cordata* L.) and soft stem bulrush (*Schoenoplectus tabernaemontani*) were used in a floating platform to remove nutrients from a stormwater retention pond [78].

Vetiver removed COD with high efficiency, and a significant difference between the vetiver system and the unplanted control setup was observed. COD removal from wastewater by plants at varying efficiencies is recognized by researchers. By using *Salvinia molesta* during phytoremediation for palm oil mill effluent, 39% of COD was removed together with the consumption of nitrogen and phosphorous [79]. In addition to 96% lead removal at an initial lead concentration of 2 mg/L, over 50% of COD was reduced by *Eichhornia crassipes* within 12 days in another study [80]. Mahajan et al. (2019) treated diluted textile effluent by phytoremediation using *Chara vulgaris*, which showed 78% of the initial COD at 216 mg/L was removed [81]. Tambunan et al. (2018) found 81% of COD can be removed from the original batik wastewater with a high initial COD at 2900 mg/L by vetiver grass; by using diluted batik wastewater, the COD removal efficiency reached 89% [67]. This high COD removal capacity by vetiver reported by Tambunan et al. (2018) is consistent with our study.

## 5. Conclusions

Our PFR system showed high efficiency and great promise in treating secondary wastewater effluent. Further investigation is needed to optimize the treatment system on a larger scale. The increase in biomass production in a constructed wetland setup could be an advantage, as the biomass can be periodically harvested to produce compost or bioethanol. In future field-scale experiments, two major parameters could be manipulated to optimize the constructed wetland: (i) the hydraulic retention time, and (ii) the plant density. As constructed wetland is a cost-effective and green technology, it can be used as a retrofit for an existing wastewater treatment plant or act independently to remove traditional and emerging contaminants. The majority of WWTPs in the United States are located in the outskirts of urban settlements or in rural areas where availability of land is not an issue. With minimal establishment cost (only for excavation and plumbing), a gravity-flow vetiver-based constructed wetland can be successfully operated to remove major contaminants from urban wastewater. A vetiver-based constructed wetland can also be installed at minimal cost in rural areas where residents have no access to a wastewater treatment plant. In many underdeveloped countries, wastewater is discharged directly to water bodies without any treatment. The same technique can also be applied in sewage canals because they are ideal plug flow reactors. The added benefit of vetiver is that under optimal growth conditions, it exhibits rapid growth. Whereas the harvested vetiver is primarily used for composting, the use of vetiver biomass through novel initiatives such as using vetiver biomass for biogas and bioethanol production could promote sustainability.

## Figures and Tables

**Table 1:** Physical and chemical characteristics of secondary wastewater effluent.

Wastewater Parameters	Values	Units
pH	6.8	
Electrical conductivity	801	μS/cm
Turbidity	13.4	NTU *
Dissolved oxygen	7.3	mg/L
Chemical oxygen demand (COD)	62	mg/L
Total nitrogen (TN)	14.4	mg/L
Total phosphate (TP)	12.3	mg/L
Total organic carbon (TOC)	21	mg/L

NTU, Nephelometric Turbidity Units.

## Data Availability

Data presented in this study are available on request from the corresponding author.

## Supplementary Materials

The following are available online at https://www.mdpi.com/article/10.3390/toxics9040084/s1, Figure S1: Removal of total nitrogen content according to predicted and experimental data from day 7 to 10, Figure S2: Removal of total phosphorus content according to predicted and experimental data from day 7 to 10.
