# Supplementary material for: Removal of Antibiotics and Nutrients by Vetiver Grass (Chrysopogon zizanioides) from a Plug Flow Reactor Based Constructed Wetland Model
Source: Toxics. 2021 Apr 15;9(4):84. doi: 10.3390/toxics9040084 (PMC8071396; doi:10.3390/toxics9040084)
Supplement: Supplementary file 1 [file toxics-09-00084-s001.zip › toxics-1158425-supplementary.pdf]

Supplementary Materials: Removal of Antibiotics and Nutrients by Vetiver Grass (*Chrysopogon zizanioides*) from a Plug Flow Reactor Based Constructed Wetland Model

Saumik Panja [1], Dibyendu Sarkar [1,*], Zhiming Zhang [1] and Rupali Datta [2]

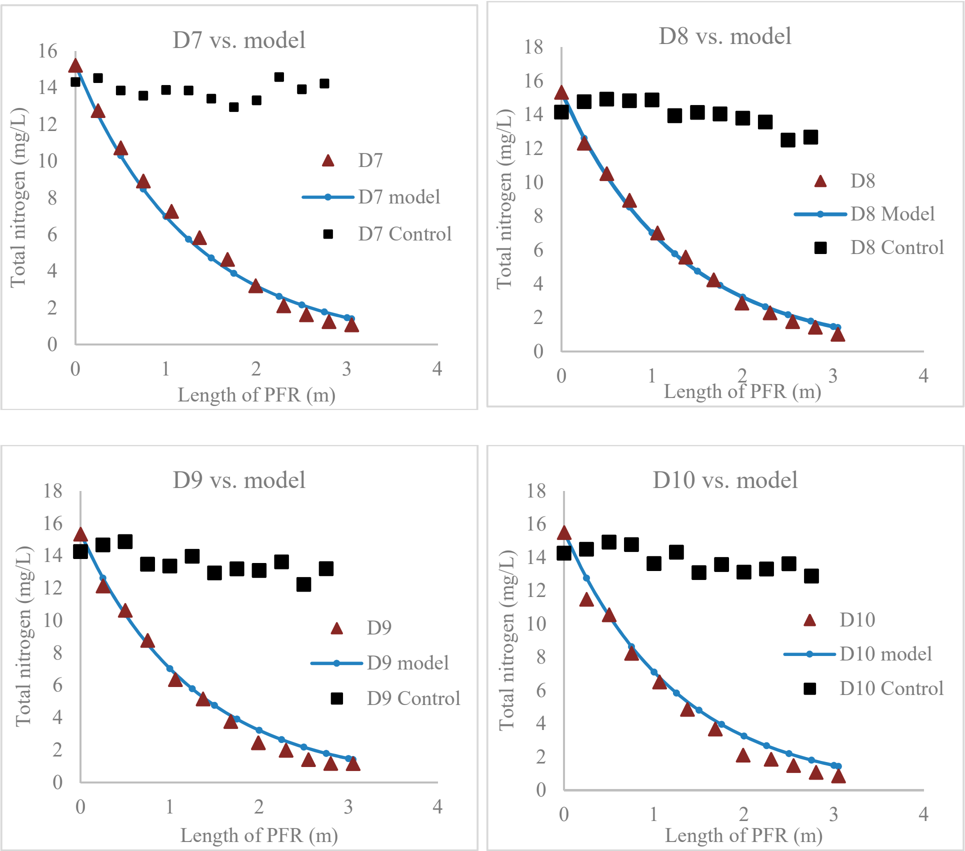

Figure S1. Removal of total nitrogen content according to predicted and experimental data from day 7 to 10.

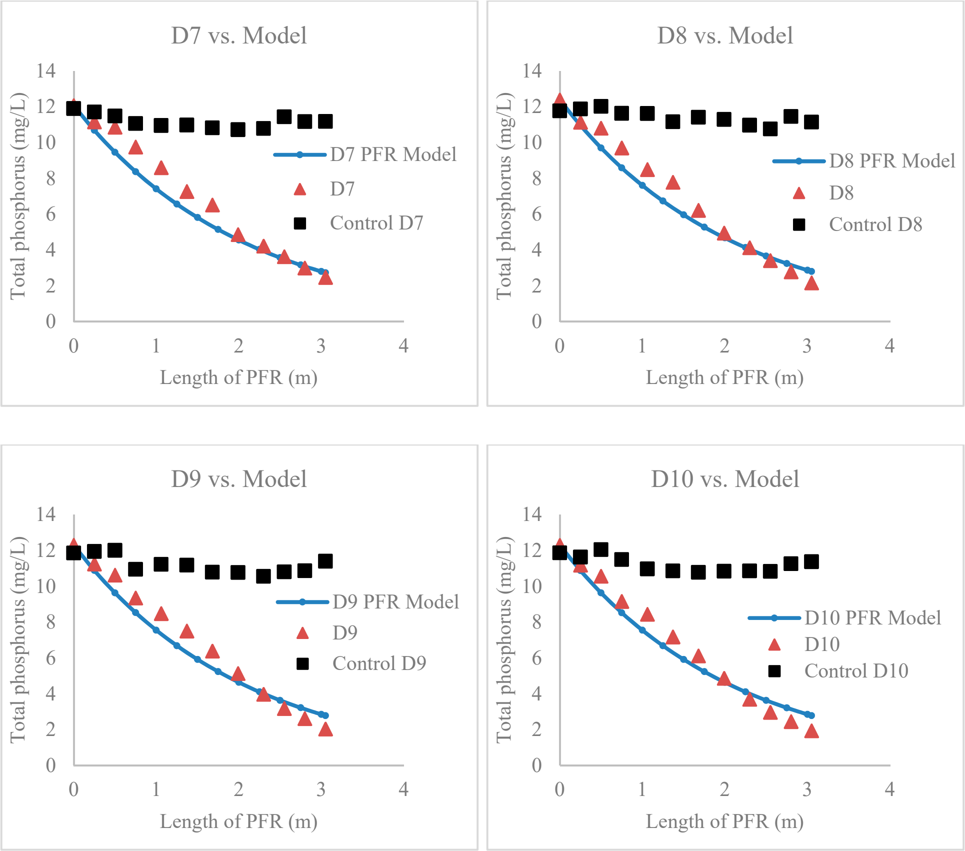

Figure S2. Removal of total phosphorus content according to predicted and experimental data from day 7 to 10.
